# Response surface methodology-based optimization of production media and purification of α-galactosidase in solid-state fermentation by *Fusarium moniliforme* NCIM 1099

**DOI:** 10.1007/s13205-016-0575-7

**Published:** 2016-12-09

**Authors:** Sanjivani B. Gajdhane, Prashant K. Bhagwat, Padma B. Dandge

**Affiliations:** 1 Department of Microbiology, Shivaji University, Kolhapur, 416004 Maharashtra India; 2 Department of Biochemistry, Shivaji University, Kolhapur, 416004 Maharashtra India

**Keywords:** α-Galactosidase, *Fusarium moniliforme*, Response surface methodology, Plackett–Burman design, Central composite rotatable design, SDS-PAGE

## Abstract

Response surface methodology was used to enhance the production of α-galactosidase from *Fusarium moniliforme* NCIM 1099 in solid-state fermentation. Plackett–Burman design was employed for selection of critical media constituents which were optimized by central composite rotatable design. Wheat bran, peptone and FeSO_4_·7H_2_O were identified as significant medium components using PB design. Further CCRD optimized medium components as wheat bran; 4.62 μg, peptone; 315.42 μg, FeSO_4_·7H_2_O; 9.04 μg. RSM methodological optimization increased the enzyme production from 13.17 to 207.33 U/g showing 15.74-fold enhancement. The α-galactosidase was purified by 70% fractionation followed by DEAE anion exchange column chromatography which yields 23.33% with 28.68-fold purification. The molecular weight of α-galactosidase was 57 kDa which was determined by SDS-PAGE analysis. Purified enzyme has optimum pH of 4.0 and was found to be stable in wide pH range of 3.0–9.0. Its optimum temperature was 50 °C, whereas its activity remains above 50% up to 2 h at 75 °C. Hg^2+^ was found to be a potent inhibitor and Mg^2+^ acted as an activator of enzyme. No significant change was observed in enzyme activity for galactose concentration, ranging from 1 to 100 mM. The *K*_m_ values of enzyme for substrates *p*-nitrophenyl-α-d-galactopyranoside, melibiose and raffinose were 0.20, 1.36, and 3.66 mM, respectively. Low *K*_m_ and stability to various physiological conditions of enzyme represents its potential which can be exploited in various industrial applications.

**Electronic supplementary material:**

The online version of this article (doi:10.1007/s13205-016-0575-7) contains supplementary material, which is available to authorized users.

## Introduction

α-Galactosidase (α-d-galactoside galactohydrolase EC. 3.2.1.22) is an exoglycosidase which catalyses hydrolysis of terminal α-1,6-linked galactosyl residue from a wide range of substrates, including oligosaccharides of raffinose family sugars, such as stachyose, melibiose, verbascose and polysaccharides of galactomannans, locust bean, and guar gum (Anisha and Prema 2006, Talbot and Sygusch 1990).

α-Galactosidase has been reported to various biotechnological and clinical applications like processing of soymilk (Tzortzis et al. 2003), conversion of blood type (Goldstein et al. 1982), and in the treatment of Fabry’s disease (Fuller et al. 2004). Commercially, α-galactosidase is used in different industries like beet sugar industry for removing raffinose from molasses and sugar syrups (Sirisha et al. 2010). Similarly, it is reported to increase the nutritional quality of legumes by hydrolyzing galacto-oligosaccharides, galactomannans, which is also responsible for improved gelling in guar gum (Bulpin et al. 1990). Due to the presence of α-galactosides like raffinose and stachyose in soybeans, humans are incapable to digest soy-derived products (Silvestroni et al. 2002). The use of microbial α-galactosidase found to be a promising solution to degrade such non-digestible oligosaccharides in soy products, which opens an avenue for its use at industrial level (Cruz and Park 1982).

Over the period, fermentation techniques have achieved immense importance due to their economic, social, and environmental advantages. Solid-state (SSF) and submerged fermentations (SmF) are two types of fermentation techniques in which SSF has gained importance for the production of microbial enzymes, due to economical advantages, such as use of cheap and abundant agro-industrial waste as a substrate. It has special interest in the processes where the crude fermented product is used directly as the enzyme source (Pandey et al. 1999). Among various groups of microorganisms used in SSF, the filamentous fungi are the most exploited because of their ability to grow on solid substrates and production of wide range of extracellular enzymes (Archana and Satyanarayana 1997). SSF is superior to SmF due to various advantages like simple media composition (cost-effective constituents), aeration process, releases negligible quantity of effluent, thus reducing pollution concerns. In addition, they efficiently provide concentrated products.

Up till now, many researchers studied α-galactosidase from various perspectives using different microorganisms. A novel α-galactosidase was identified from the hyperthermophile archaeon *Sulfolobus solfataricus* (Brouns et al. 2006). Cellular localization and purification were studied in variety of microorganisms, such as *Saccharomyces carlbergensis* (Lazo et al. 1977), *Pichia gulliermandii* (Church and Meyers 1980)*, Candida javanica* (Cavazzoni et al. 1987), *Aureobasidium pullulans* (Saha and Bothast 1998), and *Pseudomonas sp.*(Hema and Helen 2012).

Response surface methodology (RSM) is responsible for increased product formation which is the need of hour. Response surface methodological optimization can overcome the problems associated with classical optimization. RSM is an assortment of statistical techniques for designing experiments, building models, evaluating the interactive effects of variables, and pointing the optimum conditions (Shivam et al. 2009). RSM is widely used in bioprocess technology for optimization of remarkable types of fermentation media (Desai 2008, Sunitha et al. 1999). Optimization of α-galactosidase using RSM has been previously reported from various microorganisms like a mutant strain *Aspergillus foetidus* (Liu C et al. 2007), *Streptomyces griseoloalbus* (Anisha et al. 2007) and *Aspergillus niger* MRSS 234 (Srinivas et al. 1994), *Rhizopus oryzae* Strain SUK (Gajdhane et al. 2016), etc.

But till date, not even a single report is present on statistical optimization and production of α-galactosidase from *Fusarium monoliforme* NCIM 1099. Therefore, the objectives of the present work are 1. to determine the potency of *Fusarium monoliforme* NCIM 1099 for the production of α-galactosidase, 2. development of an optimal medium composition for the production of α-galactosidase in SSF using response surface methodology, and 3. enzyme extraction, purification, and characterization of purified α-galactosidase.

## Materials and methods

### Chemicals and media components

Para-nitrophenyl α-d-galactopyranoside (pNPG), raffinose, melibiose, and *p*-nitrophenol were purchased from Sigma-Aldrich (India), and all other sugars and chemical reagents were of analytical grade and procured from Himedia (India) and local suppliers.

### Culture maintenance and evaluation for the α-galactosidase activity

*Fusarium moniliforme* NCIM 1099 was obtained from the National Collection of Industrial Microorganisms, NCL, Pune (M. S.), India, and maintained on potato dextrose agar (PDA). Different agro-industrial brans, such as wheat bran, rice bran, green gram bran, cowpea bran, soybean bran, black gram bran, sunflower bran, flax seeds bran, and red kidney beans bran, were studied for their effect on the production of α-galactosidase. The production media contained 5 g of solid substrate moistened with distilled water, in 250 ml Erlenmeyer flasks initially maintained at pH 7. The media were inoculated with 1% of inoculum collected from 48 h grown culture of *Fusarium moniliforme*. Inoculated production media were incubated under static conditions at 37 °C and enzyme production was checked after every 24 h for 10 days. Enzyme was extracted in 50 ml, 0.2 M McIlvaine buffer on a rotary shaker at 180 rpm for 15 min. The content was centrifuged and the supernatant was used as the enzyme source.

### Optimization of media components for enhanced α-galactosidase production

Optimal media components for α-galactosidase production were screened by one factor-at-a-time method, keeping others constant (Liu C et al. 2007). In this experimental set-up, effect of additional carbon sources, such as glucose, galactose, fructose, maltose, lactose, xylose, arabinose, sucrose, melibiose, cellulose, and starch, were studied at 0.01 g/gds (Sirisha et al. 2010). To study the effect of different nitrogen sources on α-galactosidase production, various organic (yeast extract, peptone, soybean meal, soy peptone, and urea) and inorganic compounds (ammonium sulphate, ammonium chloride, and potassium nitrate) at 0.03 g/gds were supplemented (Shivam et al. 2009). Effect of different essential minerals on α-galactosidase production was evaluated by supplementing FeSO_4_·7H_2_O, 0.001%; CuSO_4_·5H2O, 0.05%; MgSO_4_·7H_2_O, 0.05%; NaH_2_PO_4_, 0.05%; and KH_2_PO_4_, 0.05% in production medium (Siham and Hashem 2012). All experiments were conducted in three sets and data were presented as mean values with ±SD.

### Media optimization using statistical tools

#### Plackett–Burman design

Wheat bran, peptone, FeSO_4_·7H_2_O, MgSO_4_·7H_2_O, inoculum age, inoculum size, incubation period, and pH were selected on the basis of classical method optimization studies which were further analyzed by Plackett–Burman design using Design Expert STAT Ease software 9.0 version (Minneapolis, USA). PB design was used to identify the critical media components influencing the production of α-galactosidase significantly. Each independent variable was tested for its low (−), high (+), and (0) middle values (Supplementary data Table 1). The experimental design with observed results is illustrated in Table 1. The PB design was based on the first-order model with the following equation.

1 $$Y \, = \, \beta_{\text{o}} \, + \, \varSigma \, \beta_{\text{i}} \, X_{\text{i}} , { }$$

where *Y* is the response for productivity of α-galactosidase, *β*_o_ is the intercept, and *β*_i_ is the linear coefficient. Regression analysis was used to identify critical components with significant *p* value (<0.05) influencing the production of α-galactosidase. This model is inadequate to describe the interactions occurring between the variables and only used to screen and evaluate the critical variables affecting the production of α-galactosidase. Thus, the selected variables were further optimized by CCRD of response surface methodology.

**Table 1:** Plackett–Burman experimental design representing observed and predicted responses for production of α-galactosidase enzyme

Trial	Coded value											Actual response (U/g)	Predicted response (U/g)
	*A*	*B*	*C*	*D*	*E*	*F*	*G*	*H*	*I*	*J*	*K*		
1	−1	1	1	1	−1	−1	−1	1	−1	1	1	41.26	40.98
2	−1	−1	1	−1	1	1	−1	1	1	1	−1	27.13	26.65
3	1	1	−1	1	1	1	−1	−1	−1	1	−1	53.24	52.14
4	1	−1	1	1	−1	1	1	1	−1	−1	−1	45.04	44.87
5	1	1	1	−1	−1	−1	1	−1	1	1	−1	45.8	46.76
6	−1	−1	−1	1	−1	1	1	−1	1	1	1	31.6	32.58
7	0	0	0	0	0	0	0	0	0	0	0	61	61
8	1	−1	−1	−1	1	−1	1	1	−1	1	1	32.56	31.87
9	1	1	−1	−1	−1	1	−1	1	1	−1	1	37.64	35.54
10	−1	1	1	−1	1	1	1	−1	−1	−1	1	38.53	38.69
11	−1	1	−1	1	1	−1	1	1	1	−1	−1	49.86	50.03
12	1	−1	1	1	1	−1	−1	−1	1	−1	1	41.96	40.08
13	−1	−1	−1	−1	−1	−1	−1	−1	−1	−1	−1	19.73	18.65

#### Central composite rotatable design for media optimization

Further optimization of media was done using three critical media components, wheat bran, peptone, and FeSO_4_·7H_2_O, which were selected from PB Design and were coded at five levels (−α, −1, 0, +1, +α) (Supplementary data Table 2). Design of matrix includes 20 experimental runs which consist of 12 random points, 6 center points, and 2 axial points. Three-dimensional (3D) surface plots were developed by respective data of experiments and quadratic equation was derived as shown below.

2 $$Y = \beta_{0} { + }\beta_{ 1} A{ + }\beta_{ 2} B{ + }\beta_{ 3} C{ + }\beta_{ 1} \beta_{ 1} A^{ 2} { + }\beta_{ 2} \beta_{ 2} B^{ 2} { + }\beta_{ 3} \beta_{ 3} C^{ 2} { + }\beta_{ 1} \beta_{ 2} AB{ + }\beta_{ 1} \beta_{ 3} AC{ + }\beta_{ 2} \beta_{ 3} BC ,$$

where *Y* is the response enzyme activity in units; *A*, *B*, and *C* are the coded independent variables; *β*_1_, *β*_2_, and *β*_3_ were linear coefficients; *β*_0_ was the intercept term; *β*_1_ *β*_1_, *β*_2_ *β*_2_, and *β*_3_ *β*_3_ are the quadratic coefficients; *β*_1_ *β*_2_, *β*_1_ *β*_3_, and *β*_2_ *β*_3_ are the interactive coefficients. The CCRD design was developed using Design Expert STAT Ease experimental design and was shown in Table 2.

**Table 2:** CCRD experimental design developed using Design Experts STAT Ease Software

Trial	Coded value			Actual response (U/g)	Predicted response (U/g)
	*A*	*B*	*C*		
1	0	0	0	189.21	189.41
2	α	0	0	175.75	174.24
3	0	0	α	179.14	177.63
4	1	1	1	178.63	178.02
5	1	−1	1	174.33	177.17
6	0	0	0	186.76	189.41
7	0	0	0	189.17	189.41
8	−α	0	0	150.52	149.92
9	−1	−1	−1	151.34	153.42
10	0	0	0	188.44	189.41
11	0	0	0	192.33	189.41
12	0	−α	0	163.08	162.55
13	1	1	−1	160.34	163.24
14	−1	−1	1	154.45	153.02
15	0	0	0	190.22	189.41
16	0	α	0	169.08	167.51
17	1	−1	−1	171.21	169.79
18	0	0	−α	166.14	165.54
19	−1	1	−1	159.84	158.47
20	−1	1	1	162.57	165.47

### Analytical techniques

#### Extraction of enzyme

For enzyme extraction, a known quantity of the fermented matter was mixed with McIlvaine buffer 30 ml (pH 7) by shaking on a rotary shaker (180 rpm, 15 min) and centrifuged at 10,000×*g* for 10 min (4 °C) and the supernatant was used as crude enzyme.

#### Enzyme assay

Assay of α-galactosidase was carried out by the method of Dey with some modifications (Dey and Wallenfels 1974). One milliliter of reaction mixture contains 0.2 ml of enzyme, 0.2 ml of 0.02 M citrate phosphate buffer pH 5.0, and 0.6 ml of 1 mM *p*-nitrophenyl α-d-galactopyranoside (*p*NPG) solution in the same buffer. The mixture was incubated for 10 min at 50 °C and the reaction was arrested by the addition of 3 ml of 0.5 M sodium carbonate solution. The amount of *p*-nitrophenol liberated was measured by the absorbance at 405 nm. One unit of enzyme activity was defined as the quantity of enzyme that liberated 1 µmol of *p*-nitrophenol per min per ml of enzyme under assay conditions. The specific activity was articulated as units of enzyme activity per mg of protein content.

#### Purification of enzyme

The extracellular enzyme was subjected to 70% ammonium sulphate precipitation. The precipitate was collected by centrifugation at 8000 rpm for 15 min at 4 °C then re-dissolved in 10 ml 0.02 M citrate phosphate buffer of pH 5.0, and dialyzed against the same buffer. The dialyzed enzyme was concentrated and subjected to DEAE cellulose, previously equilibrated with McIlvaine buffer (20 mM, pH 4.0). The enzyme was eluted with linear gradient of 0.1–0.5 M NaCl at a flow rate of 20 mlh^−1^ and fractions of 5 ml were collected. All the fractions were checked for α-galactosidase activity and protein content was measured at 280 nm. The active fractions having high α-galactosidase activity were pooled, stored at 4 °C, and used for further enzyme study. Lowry method was used for the protein quantification and bovine serum albumin was used as standard (Lowry et al. 1951). Sodium dodecyl sulphate-polyacrylamide gel electrophoresis (SDS-PAGE) was performed according to earlier protocol (Phugare et al. 2011) with 4% stacking gel and 12% separating gel using a vertical gel electrophoresis system (GeNei, Bangalore, India). Protein bands were visualized by silver staining and protein marker (6.4–205 kDa, Genei, India) were used for molecular weight determination of purified α-galactosidase.

### Characterization of α-galactosidase

#### Effect of pH and temperature on enzyme activity and stability

To estimate the optimum pH for α-galactosidase activity, the various buffers were used in varying pH ranges like citrate phosphate buffer (for pH 3–6), phosphate buffer (for pH 7–8), and glycine-NaOH buffer (for pH 9–11), at 50 °C. Its pH stability was studied by pre-incubation in buffer solutions (pH 3.0–10.0) for 24, 48, and 72 h at 50°C. Aliquots were withdrawn and assayed for residual α-galactosidase activity. Residual activity was expressed as percentage of activity as compared to untreated enzyme. The effect of temperature on the enzyme activity was evaluated using different incubation temperatures ranging from 10 to 80 °C and keeping other physicochemical parameters unaltered. Thermostability of purified enzyme was determined by pre-incubating the enzyme at 0, 55, 60, 65, 70, and 75 °C for a time interval of 30–210 min. In each of the panels, the activity under optimum condition was set as 100%.

#### Effect of different metal ions, reagents, galactose, and organic solvent on enzyme

The effect of different metal ions, such as Cu^2+^, Mg^2+^, Ca^2+^, Co^2+^, Ba^2+^, and Hg^2+^, each at 1 mM concentration on enzyme activity was studied. Similarly reagents like EDTA, β-mercaptoethanol, and urea (each at 1 mM) were also studied for their effect on enzyme activity. To check the feedback inhibition, different concentrations of galactose (1–100 mM) incubated with purified enzyme for 30 min at 37 °C and further assayed under standard conditions. Organic solvents tolerance (for methanol, butanol, *n*-propanol, glycerol, and ethyl alcohol) of the enzyme was investigated by determining its residual activity under standard assay conditions after incubating enzyme with organic solvent in 1:1 ratio for the period of 1–6 days, respectively.

#### Enzymatic hydrolysis of melibiose

To 500 μl of 1% solution (w/v) of melibiose in 20 mM citrate phosphate buffer (pH 4.0), 100 μl of α-galactosidase (32 U) was added. Mixtures were incubated at 50 °C and analyzed by TLC on silica plates using n-butanol/ethanol/water (10:8:7) as a solvent system. Sugars were detected by spraying the dried chromatograms with 1% orcinol (w/v) in ethanol containing 10% sulfuric acid (v/v).

#### Substrate specificities and kinetic parameters

The relative substrate specificities of the α-galactosidase towards synthetic (pNPG) and natural substrates (Melibiose and Raffinose) were determined under standard assay conditions. For kinetic studies, the initial rate of hydrolysis of various glycosides at different concentrations (pNPG: 1–10 mM, Melibiose and Raffinose: 1–20 mM) was measured under standard assay conditions, and the kinetic constants *K*_m_ and *V*_max_ were determined from Lineweaver–Burk plot. All data obtained from the enzyme kinetic assays and Microsoft Office Excel were used for plotting.

#### Statistical analysis

Obtained results were mean of three determinants. Microsoft Office data’ analysis tool pack was used to do ANOVA. Difference of *p* ≤ 0.05 was considered as significant.

## Results

### Classical optimization of various parameters for enzyme production

Among different substrates used, wheat bran was the suitable substrate in SSF with 13.17 U/g activity (Supplementary data Fig. 1). To study the effect of pH of the medium on enzyme production, experiments were performed with media of different pH and incubated for 96 h. Enzyme production progressively increased with increase in pH of the medium from 5.0 to 7.0 and maximum production (13.44 U/g) was recorded at pH 7.0. The enzyme yield decreases if the medium pH was higher than this. In fermentation, size of inoculum is an important biological factor which determines biomass production. Maximum enzyme production was obtained when SSF medium was inoculated with 1% of spore suspension. Enzyme production yield was optimum with 55% of moisture level which is a critical factor of SSF. Maximum enzyme production was 16.55 U/g following the study of all physiological parameters.

### Effect of carbon and nitrogen sources on α-galactosidase activity

The carbon source employed in microbial enzyme production is one of the most important factors of SSF. In this study, cultivations were performed with wheat bran along with additional carbon sources. Combinational effect of wheat bran with that of pure carbon sources does not showed any amendment in enzyme production. Hence, wheat bran itself fulfills the requirement of carbon source and also acts as most significant substrate; hence, further experiments were carried out with it (Supplementary data Fig. 2).

The type of nitrogen source is equally important as a nutritional requirement of organism. The effects of organic and inorganic nitrogen sources were further studied at constant carbon source (wheat bran). In this study, peptone was found to be the most effective for α-galactosidase production (25.49 U/g) which was followed by yeast extract (23.47 U/g), meat extract (23.60 U/g), and soybean meal (23.60 U/g), while inorganic nitrogen sources failed to increase the α-galactosidase production (Supplementary data Fig. 3).

### Effect of minerals on enzyme production

Apart from carbon and nitrogen sources, many other essential minerals, such as magnesium, calcium, and trace elements, are required to support active cellular function. Accordingly, an effect of minerals on the production of α-galactosidase was studied with the inclusion of best carbon and nitrogen sources in the production medium. Results demonstrated that FeSO_4_·7H_2_O (28.15 U/g) was the most effective mineral inducing α-galactosidase activity which was followed by MgSO_4_·7H_2_O (26.6 U/g), whereas KH_2_PO_4_ (25.3 U/g), NaH_2_PO_4_ (25 U/g), MnSO_4_·H_2_O (24.7 U/g), CuSO_4_·5H_2_O (23.14 U/g), and CaCl_2_·2H_2_O (16.52 U/g) showed negative effect on α-galactosidase production.

### Statistical medium optimization studies

#### Plackett–Burman design

Exploration of the most effective nutrient components influencing the yield of α-galactosidase production was carried out using PB design. Among eight investigated variables, three variables, viz., wheat bran, peptone, and FeSO_4_·7H_2_O, were enmarked as the most effective ones influencing the yield of enzyme with their significant *p* values where the values of “Prob > *F*” less than 0.05 specify model terms are significant, while remaining components show *p* values (0.1) which are above the significant level; hence, those components are considered to be insignificant.

The main effect of medium components, standard analysis of variance (ANOVA), regression coefficient, *F* values, and *p* values of variables examined in this study, and are illustrated in Table 3. The model *F* value 18.67 implies that the model is significant and there is only 0.06% chance that this large value could occur due to noise; in addition, the pre-determined *R* ^2^ 0.72 was in reasonable agreement with the adjusted *R* ^2^ 0.83. The adequate exactitude measures signal-to-noise ratio. A ratio greater than 4 is desirable and 12:68 ratio indicated an adequate signal; thus, this model can be used to navigate the design space. The final equation was derived in terms of actual factors which revealed the yield of enzyme as function of independent variables represented as follows:

**Table 3:** ANOVA for first-order model in PB Design

Source	Sum of squares	*df*	Mean square	*F* value	*p* value > *F*
Model	972.18	3	324.06	18.67	0.0006
A-wheat bran	155.02	1	155.02	8.93	0.0174
B-peptone	447.86	1	447.86	25.81	0.0010
D-FeSO4	369.30	1	369.30	21.28	0.0017
Residual	138.83	8	17.35		
Corrected total	1111.01	11			

3 $${\text{Enzyme activity (units) }} = { 38}. 2 8 { } + { 3}. 5 9A \, + { 6}. 1 1B \, + { 5}. 5 5D .$$

Using PB experimental design, only effective critical components affecting enzyme production were identified, but it does not examine the interactive effects between selected critical components which were further studied by employing central composite design (CCRD) of RSM.

### Central composite rotatable design

The CCRD design experiment was developed using three factors at two-level factorial. Based on the quadratic regression analysis, the CCRD results substantiated the subsequent second-order polynomial equation. The equation is as follows,

4 $${\text{Enzyme activity (U}}/{\text{g}}) \, = { 189}. 4 1 { } + { 7}. 2 3A \, + { 1}. 4 7B \, + { 3}. 60C \, - { 2}. 90AB \, + { 1}. 9 5AC \, + { 1}. 8 5BC \, - { 9}. 6 6A^{ 2} - { 8}. 6 2B^{ 2} - { 6}. 30C^{ 2} .$$

The interactive effects of medium components deduced by standard analysis of variance (ANOVA), regression coefficient, *F* values, and *p* values of variables were examined, and are illustrated in Table 4. The model *F* value 65.10 implies that the model is significant and there is only 0.1% chance that model *F* value, this large could occur due to noise. The values of “prob > *F*” less than 0.05 showed model terms (*A*, *C*, *AB*, *AC*, *A* ^*2*^, *B* ^2^, and *C* ^2^) are significant and values greater than 0.1 indicated model terms are nonsignificant. The pre-determined *R* ^2^ 0.89 was in reasonable agreement with the adjusted *R* ^2^ 0.97 which depicted adequacy of model to predict response. Adequate precision measures signal-to-noise ratio, precision ratio greater than four is desirable, and the ratio is 22:64. Therefore, model can be used to navigate the design space. The “Lack of Fit *F* value” of 2.52 implies that the lack of fit is not significant relative to the pure error. Non-significant lack of fit is good which confirmed that the model equation was adequate to predict the enzyme yield. The value of coefficient of variation (CV% = 1.42) revealed the precision and reliability of the model.

**Table 4:** Analysis of variance (ANOVA) for the experimental results of CCD, a Quadratic Model

Source	Sum of squares	*df*	Mean square	*F* value	Prob > *F*
Model	3562.188358	9	395.7987064	65.10166513	1.08261E−07
A-wheat bran	713.9206388	1	713.9206388	117.4269183	7.57711E−07
B-peptone	29.70304028	1	29.70304028	4.88560814	0.051530892
C-FeSO_4_·7H_2_O	176.6231711	1	176.6231711	29.05128883	0.000305755
AB	67.2220125	1	67.2220125	11.05679447	0.007680103
AC	30.3031125	1	30.3031125	4.984309071	0.049626858
BC	27.3430125	1	27.3430125	4.497426633	0.059951519
A^2^	1345.394929	1	1345.394929	221.2929166	3.78672E−08
B^2^	1071.052911	1	1071.052911	176.1686606	1.12596E−07
C^2^	572.2779435	1	572.2779435	94.12927945	2.09596E−06
Residual	60.79701734	10	6.079701734		
Lack of fit	43.57166734		8.714333468	2.52950839	0.165704499
Pure error	17.22535		3.44507		
Corrected Total	3622.985375				

*p* values are significant at *p* ≤ 0.05

### Interaction of variables

**Fig. 1:**
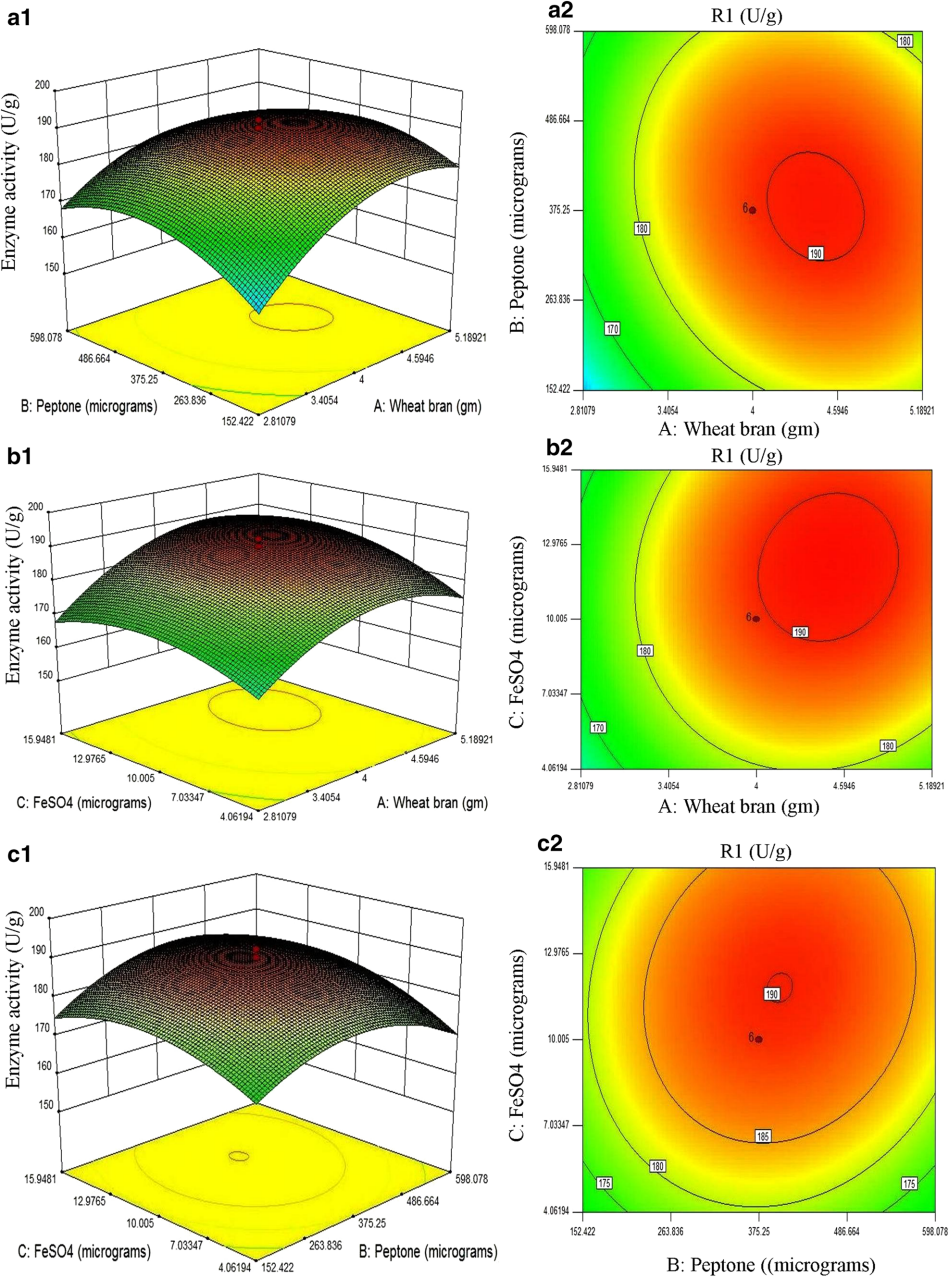
Representing the 3D surface as well as contour plots; **a1**, **a2** revealed nearly elliptical contour plots which suggested that interactions between wheat bran and peptone are moderate significant, **b1**, **b2** illustrated nearly circular interactions of FeSO_4_ and wheat bran which revealed that interactions among FeSO_4_ and wheat bran are less significant, while **c1**, **c2** revealed nearly highly elliptical contour plots which indicated that interactions among FeSO_4_ and peptone are most significant (*R*1 enzyme activity)

Graphical representations of a regression equation [three-dimensional (3D) response surface plots and their respective two-dimensional (2D) contour plots] are simple. They provide a crucial contribution for understanding the interactions between the two variables and finding their optimum levels (Jhample et al. 2015). The circular order of counter plots indicates that the mutual interactions among corresponding variables are non-significant, while the elliptical order specifies significant interactions between corresponding variables (Bhagwat et al. 2015). Two interacting variables were kept at optimum levels and the remaining variables were kept at zero level. The resulted contour and 3D response surface plots can be used to predict the yield of enzyme at different concentration of variables. 3D response surface plots and contour plots are verified in subfigures of Fig. 1. Interactions between wheat bran and peptone were showed in Fig. 1a1 and a2 which were moderate significant with nearly elliptical order of contour plots indicating their influence on yield of enzyme. When concentration of both sources increases, the enzyme yield was also considerably increased but further increase in concentration resulted in reduction of enzyme yield. In Fig. 1b1 and b2, contour plots displayed nearly circular in nature; hence, the interactions between FeSO·7H_2_O and wheat bran have less significant effect on enzyme production, while the contour plot in Fig. 1c1 and c2 showed highly elliptical nature which suggests most significant interactions among FeSO·7H_2_O and peptone.

The optimum conditions for the maximum production of α-galactosidase were determined by the response surface analysis and regression equation. The predicted values of the regression equation closely agreed with the experimental values. Experimental model was validated by testing batch experiments under optimal operating conditions. Results of three repeated experiments were compared. The α-galactosidase activity obtained from the experiments was very close to the response predicted by the regression model proving the validity of the model. At these optimized conditions, the maximum α-galactosidase activity was found to be 192.33 U/g.

### Experimental model validation

The processed parameters were set to achieve a maximum response involving optimum concentration of variables: wheat bran; 4.62 μg, peptone; 315.42 μg, FeSO_4_·7H_2_O; 9.04 μg; at these optimized conditions, the maximum α-galactosidase activity was found to be 207.33 U/g.

### Purification of α-galactosidase

**Fig. 2:**
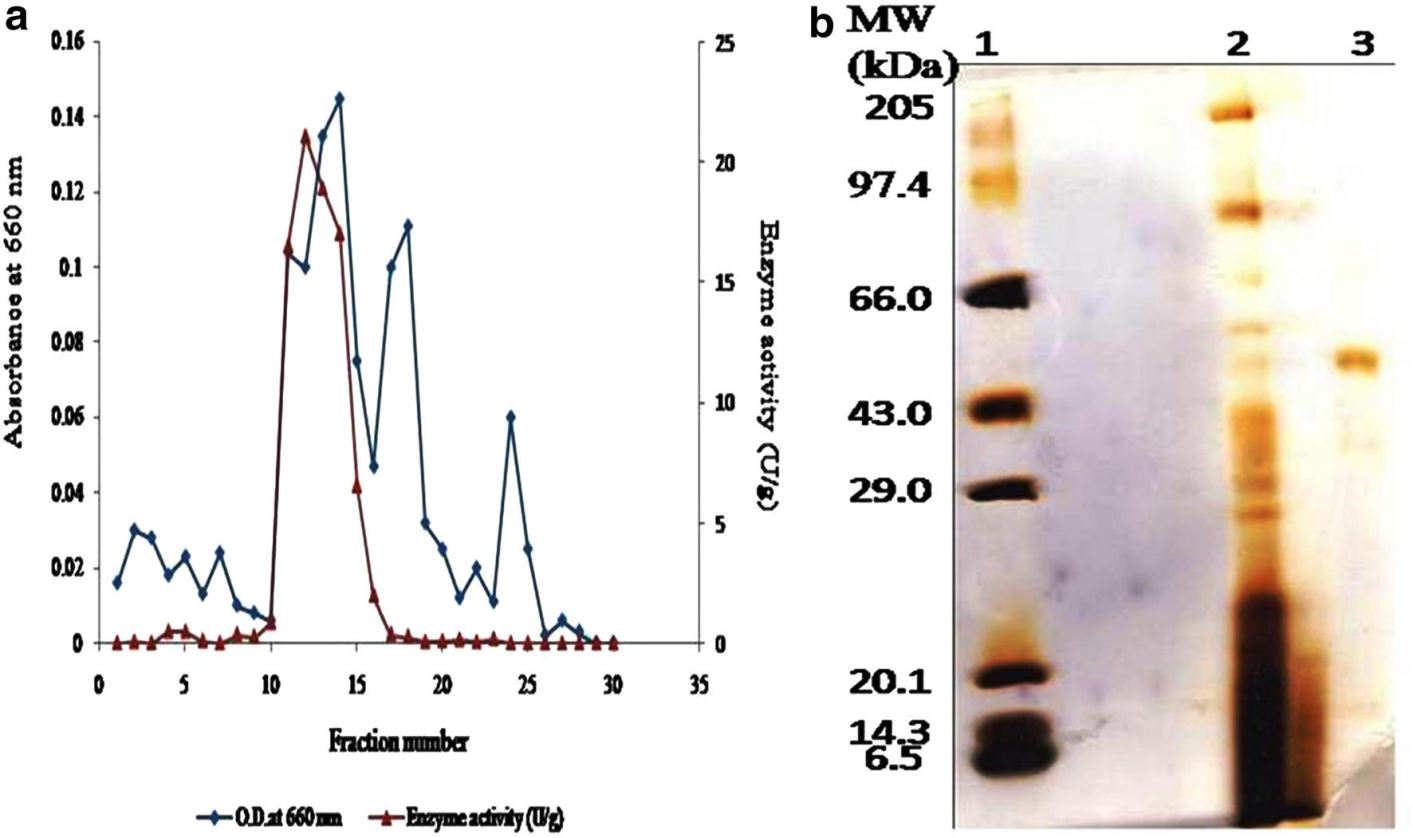
**a** Purification profile of α-galactosidase. The extracellular α-galactosidase from *Fusarium moniliforme* NCIM1099 was precipitated by 70% saturation of ammonium sulphate, desalted, concentrated, and then loaded on DEAE cellulose DE52, anion exchange column chromatography (column diameter height = 1.2 cm × 18 cm). The proteins from column were eluted at 0.1–0.5 M NaCl gradient and the protein content was determined by taking absorbance at 280 nm; α-galactosidase activity was determined by standard assay method. The fraction size was of 5 ml each. The activity has eluted in *fraction number* 12 and 13 with 0.2 M NaCl. **b** Molecular weight determination of α-galactosidase through SDS PAGE analysis; *Lane 1*, protein molecular weight markers; *Lane 2*, crude culture supernatant *Lane 3*, purified α-galactosidase after anion exchange column chromatography

The α-galactosidase from *Fusarium moniliforme* was purified to homogeneity with 28.68-fold and 23.33% yield (Table 5). The elution pattern is shown in Fig. 2a. The enzyme applied for molecular weight determination using SDS-PAGE showed a single band confirming its purity with molecular weight of 57 kDa (Fig. 2b).

**Table 5:** Purification of α-galactosidase

Enzyme fraction	Volume (ml)	Protein conc. (mg)	Activity (units)	Specific activity (U/mg)	Purification (fold)	Yield (%)
Culture filtrate	100	51.6	300	5.81	1	100
Ammonium sulphate precipitation	10	2.77	230	83.03	14.29	76.66
DEAE-cellulose chromatography	7	0.42	70	166.66	28.68	23.33

### Effect of pH and temperature on enzyme activity and stability

**Fig. 3:**
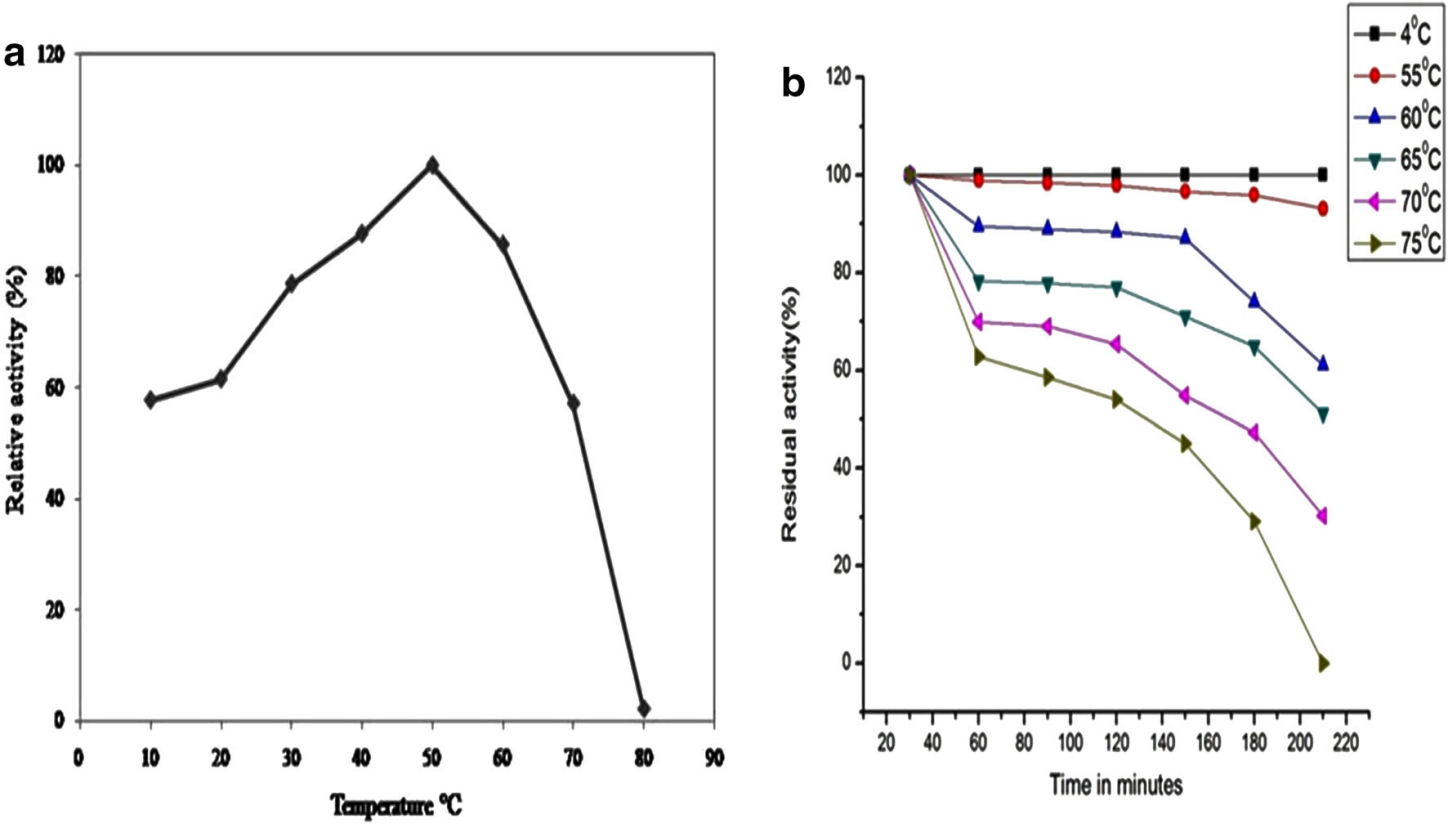
**a** Relative activity of enzyme incubated at different temperature. **b** Residual activity of enzyme incubated at different temperature for 0, 30, 60, 90, 120, 150,180, and 210 min

Purified α-galactosidase is assayed for relative and residual activity at varying pH and temperature values, keeping other parameters constant. The optimum pH of enzyme was found to be 4.0, and in case of stability, enzyme was stable at wide pH range of 3.0–9.0 (Supplementary data Fig. 4a, 4b). The relative activities at pH 3 and 11 were 94.48 and 68.40%, respectively. The enzyme retained 100% activity when preincubated for 72 h in pH 4.0 at 50 °C, whereas it retained 48.29% activity when kept in buffer of pH 10. Enzyme was having temperature optima of 50 °C (Fig. 3a), whereas it was stable from 55 to 70 °C, while it showed more than 50% of residual activity when preincubated at 70 °C for nearly 3 h. Figure 3b shows the temperature-activity profile for α-galactosidase.

### Effect of different metal ions, reagents, galactose, and organic solvents on enzyme

Among all the tested minerals, Mg^2+^ enhances the enzyme activity and it was unchanged with, Ca^2+^, Ba^2+^, K^+^, and Na^+^, whereas 100% activity was lost by Hg^2+^, indicating it as a strong inhibitor of α-galactosidase. Cu^2+^ and urea had a little inhibitory effect on enzyme, while EDTA and β-mercaptoethanol did not affect enzyme activity at all. The galactose tolerance of the enzyme was determined by estimating relative activity under standard assay conditions which was 91.94% for 100 mM galactose concentration (Supplementary data Table 3). In organic solvent stability study, relative activity of enzyme after 144 h of incubation was 99.78 and 93.6% for acetone and methanol, respectively, while glycerol had 79.75% (Supplementary data Table 4).

### Enzymatic hydrolysis of melibiose

**Fig. 4:**
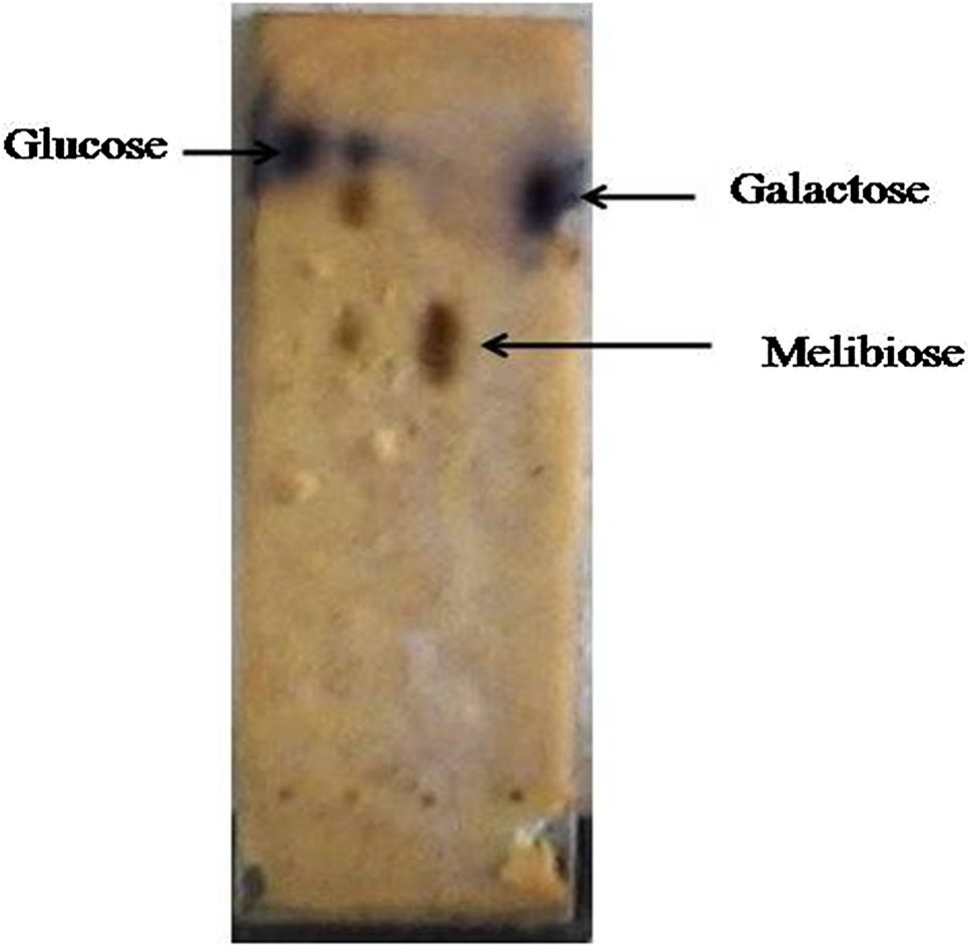
Thin-layer chromatography plate showing hydrolysis by *Fusarium moniliforme* leading to the accumulation of glucose and galactose

Hydrolysis of melibiose was examined by thin-layer chromatography. The melibiose was digested for 20 min with purified enzyme suggesting efficiency of enzyme to hydrolyze non-digestible oligosaccharide that is melibiose. It was observed that melibiose hydrolyzed to glucose and galactose (Fig. 4).

### Determination of kinetic parameters

For determination of *K*_m_, the effect of substrate concentration on the rate of hydrolysis of *p*-nitrophenyl-α-d-galactopyranoside, melibiose, and raffinose was investigated with standard assay procedure. The apparent Michaelis–Menten constants (*K*_m_) of the enzyme for pNPG, melibiose, and raffinose were 0.20, 1.36, and 3.66 mM, and maximum velocities (*V*_max_) were 1.0, 0.76, and 1.42 μmol/min/mg, respectively (Supplementary data Fig. 5).

## Discussion

Solid-state fermentation is cheap, simple, and cost-effective technique, and due to all these advantages, it may be used for production of many novel industrial products (Pandey et al. 1999, Archana and Satyanarayana 1997, Maurice 1998, Viniegra-Gonzalez et al. 2003, Beniwal 2010). The pH of the medium drastically affects the production of enzymes, but in this study, we observed that no significant difference was found in the enzyme yield with the fluctuation of pH of media (data not shown). This could be explained by the fact that wheat bran possesses excellent buffering capacity for the media (Pandey et al. 1999). Similar observations were made by Nampoothiri et al. for chitinase production in wheat bran-based SSF medium (Nampoothiri et al. 2004). In general, fungal cultures are habituated to grow at low moisture content equally in this study. *Fusarium moniliforme* showed maximum enzyme production at 55% of moisture level. The metabolic activities of the culture and consequently product synthesis were drastically affected at lower and higher moisture levels. Lower moisture leads to reduced solubility of the nutrients, whereas a lower degree of substrate swelling and higher water tension occurred (Bhatti et al. 2007). Similarly, higher moisture contents were reported to cause decreased in porosity, gas exchange capacity, loss of particle structure, development of stickiness, and reduction in gas volume (Lonsane et al. 1985).

In the current study, it was observed that wheat bran itself acted as a best carbon source, while peptone acted as a nitrogen source. In case of α-galactosidase from *Aspergillus foetidus,* wheat bran and soybean meal were reported as the best sources of carbon and nitrogen, respectively (Liu et al. 2007). Furthermore, Fe^2+^ was potent enhancer for enzyme production, followed by Mg^2+^ in our study, whereas, in case of α-galactosidase from *Aspergillus parasiticus* MTCC-2796, its activity was found to be enhanced by Mn^2+^ and Mg^2+^ (Shivam et al. 2009). In our study, decrease in the production of α-galactosidase was observed in the presence of Ca^2+^; similar results were observed by Liu CQ in case of α-galactosidase from *Aspergillus foetidus,* where Ca^2+^ decreased the production which was due to its inhibitory effect on mycelial growth and proliferation (Liu et al. 2007).

It is noteworthy that using the simplest carbon and nitrogen sources, we have succeeded in optimizing the media components using RSM giving improved enzyme yield. The experimental results of PB stated that wheat bran, peptone, and FeSO_4_·7H_2_O were critical medium components, and significantly influenced the production of enzyme. Media optimization studies were carried out using CCRD where critical medium components were further optimized and were investigated for significant influence on the yield of enzyme through interactions among them. Results implicated that interactions between wheat bran and peptone were most significant ones followed by wheat bran and FeSO_4_·7H_2_O, whereas interactions between peptone and FeSO_4_·7H_2_O were less significant. Model evaluation revealed that the experimental and predicted values were very close, indicating that experimental design is effective for process optimization. Using PB design and CCRD of response surface methodology, the yield of α-galactosidase was considerably enhanced from 13.17 to 207.33 U/g which was significantly higher as compared to *Streptomyces griseoloalbus* which showed maximum activity of 117 U/g in a RSM optimized media (Anisha et al. 2007).

In the current study, enzyme was purified with 28.68 purification fold and 23.33% yield. In earlier reports, white rot fungus *Pleurotus florida* gave only 0.17% yield with 36-fold purification (Ramlingum et al. 2007). Similarly, *Penicillium purpurogenum* gave 2% yield with purification fold of 9.44 (Ramlingum et al. 2010). Molecular weight of purified α-galactosidase in our study was found to be 57 kDa. Puchart et al. reported α-galactosidase with similar molecular weight from *Thermomyces lanuginosus* (Puchart et al. 2000). Experimental results substantiated that enzyme could work at broad range of pH and temperature. The pH stability profile indicated that the purified enzyme was stable in the pH range of 3.0–9.0 but underwent a decrease in activity above 10. Maximum α-galactosidase activity was found in the acidic pH range and decreases in the alkaline range (Rodriguez et al. 2008). The effect of pH on the activity of α-galactosidase may be attributed to its amino acids and active site which undergoes protonation or deprotonation and the conformational changes induced by amino-acid ionization (Sabu et al. 2005). Similarly, α-galactosidase from white rot fungus *Pleurotus florida* and *Monascus pilosus* was stable from pH 4.0 to 9.0, and pH 3.0 to 8.0, respectively (Wong et al. 1986). Till date, values reported on temperature stability of enzyme were up to 65 °C, but in our study, it was up to 75 °C which increases the applicability of this enzyme. However, thermostability of our enzyme was low as compared to that of α-galactosidases from extremophilic marine bacteria *Thermotoga neapolitana* (King et al. 1998) and *Thermotoga* maritime (Comfort et al. 2007). d-galactose is reported as a strong inhibitor of α-galactosidase, but our enzyme showed tolerance to d-galactose up to 100 mM concentration, similar results were reported for α-galactosidase from *Streptomyces griseoloalbus* (Anisha GS 2007), and is an important from industrial point of view, while galactose tolerance is an significant character which improves the effectiveness of α-galactosidases in liberating galactose residues from oligomeric and polymeric α-galactosides.

Activity of enzyme was completely inhibited by Hg^2+^, similar reports were observed in case of α-galactosidase from *Cucurbita pepo* plant and various fungal sources like *Torulaspora delbrueckii* IFO, *G. lucidum,* and white rot fungus *Pleurotus florida* (Gaudreault and Webb 1983, Oda and Tonomura 1996, Ramlingum et al. 2007). It was observed that EDTA has no negative effect on enzyme activity, while on the other hand, it showed inhibitory effect on *Cucurbita pepo* (Gaudreault and Webb 1983). It was interesting to note that α-galactosidase was stable in all organic solvents used, while stability was found to be maximum in acetone. Similar results were reported for α-galactosidase from *Talaromyces flavus* (Simerska et al. 2003).

Earlier, it is reported that the activity of α-galactosidase is inhibited by the higher concentrations of substrate *p*-nitrophenyl-α-d-galactopyranoside (Wong et al. 1986); in contrast to these results, in our study, there is no any inhibitory effect which was found with high concentration of pNPG. While studying the kinetic parameters, it was found that *K*_m_ for pNPG, melibiose, and raffinose were 0.20, 1.36, and 3.66 mM, respectively. α-galactosidase from both the *Monascus pilosus* as well as Cicer showed *K*_m_ of 0.8 mM for pNPG (Wong et al. 1986, Singh and Kayastha 2012), whereas it was around 1.07 for tomato α-galactosidase (Okutucu et al. 2010) and 0.27 for *Humicola* sp. α-galactosidase (Kotwal et al. 1999). In case of *Aspergillus oryzae* and *Aspergillus niger*, both of them showed *K*_m_ of 5.5 mM for raffinose (Kulkarni et al. 2006), while in case of *Humicola* sp., it was 1.45 mM for raffinose (Kotwal et al. 1999). This study revealed that our enzyme has high affinity for synthetic substrate than natural substrate raffinose which is a larger and more bulky trisaccharide. Melibiose was hydrolyzed to glucose and galactose by enzyme under study, and was proved by TLC analysis, similar results were reported by α-galactosidase from *Azotobacter vinelandii* (Wong 1990).

## Conclusion

Microbial enzymes are more efficient, significant, cheap, and easily available as compared to plants and animal enzymes. Therefore, nowadays, industrially applicable microbial enzyme production is thirst area of research. Our present investigation emphasized on production of α-galactosidase from potent microbe *Fusarium moniliforme* NCIM 1099 by response surface methodology in solid-state fermentation. It is significantly noted that statistically optimized medium with simplest carbon and nitrogen sources showed noteworthy increase in the production of α-galactosidase enzyme. The extracellular α-galactosidase was isolated from *Fusarium moniliforme* NCIM 1099 and was purified to its highest purity. Considering the characteristics of α-galactosidase produced from *Fusarium moniliforme* NCIM 1099, such as ability to hydrolyze non-digestible sugars, broad pH, temperature activity, and stability; opens an avenue for its use in various food industries; thus this strain could be an excellent candidate for the industrial production of α-galactosidase.

## Electronic supplementary material

Below is the link to the electronic supplementary material.

Supplementary material 1 (DOCX 704 kb)
