# Supplementary material for: Response surface methodology-based optimization of production media and purification of α-galactosidase in solid-state fermentation by Fusarium moniliforme NCIM 1099
Source: 3 Biotech. 2016 Dec 9;6(2):260. doi: 10.1007/s13205-016-0575-7 (PMC5148754; doi:10.1007/s13205-016-0575-7)
Supplement: Supplementary file 1 — Supplementary material 1 (DOCX 704 kb) [file 13205_2016_575_MOESM1_ESM.docx]

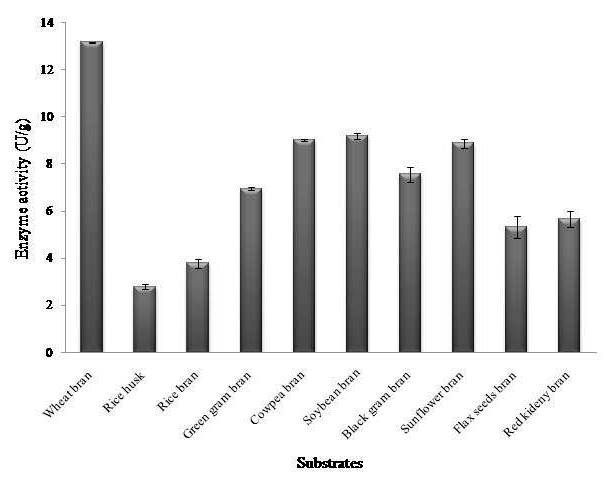


**Fig. 1** Use of different substrates for production of α-galactosidase


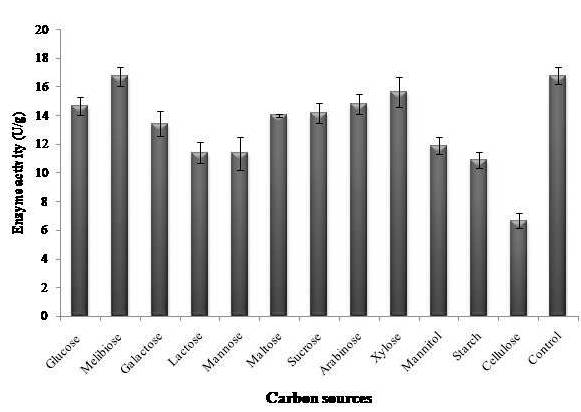


**Fig.2** Different carbon sources used in production of enzyme


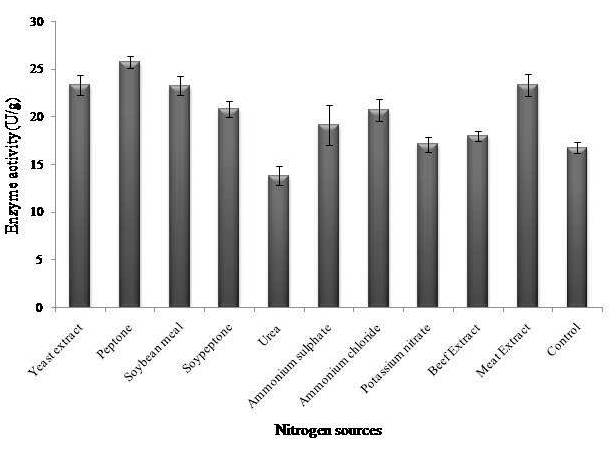


**Fig. 3** Different nitrogen sources used in production of enzyme


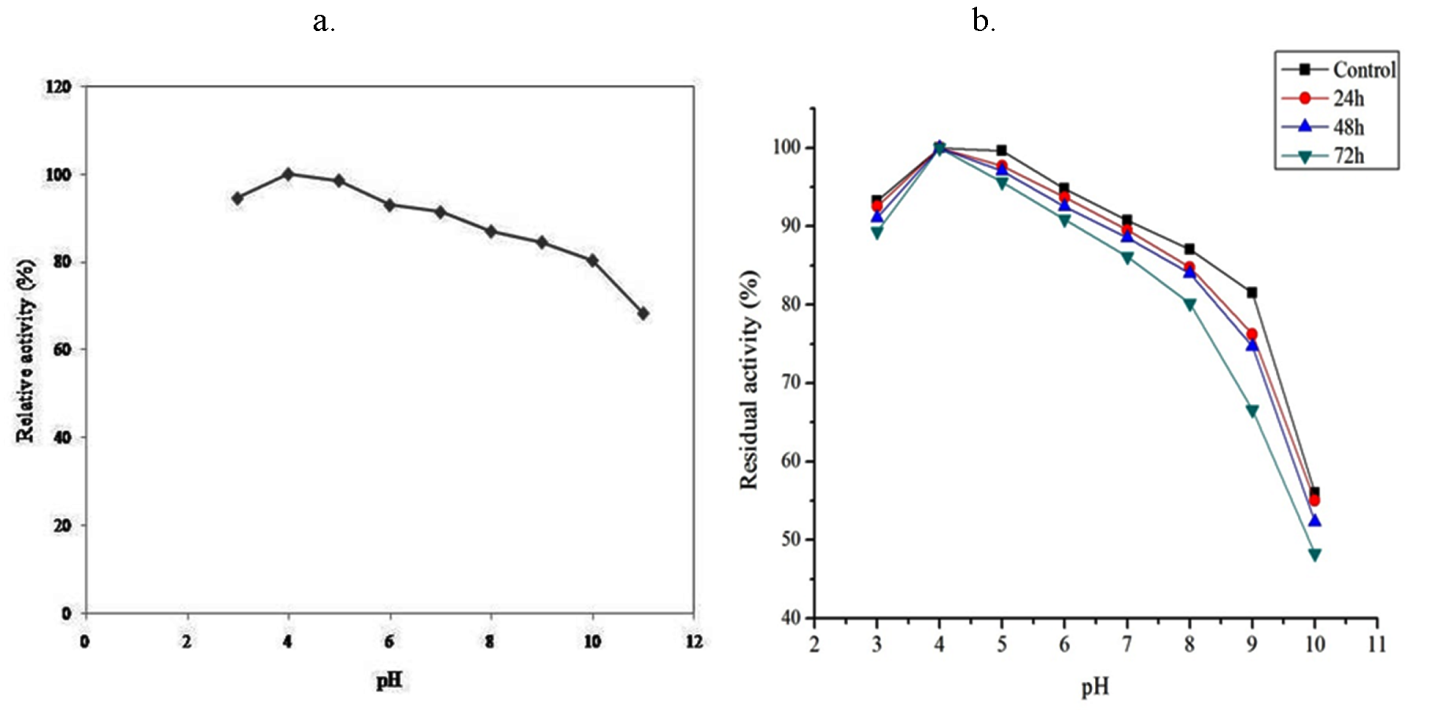
 **Fig.4 (a)** Relative activity of enzyme incubated at different pH **(b)** Residual activity of enzyme incubated at different pH for control, 24 h, 48 hand 72 h.


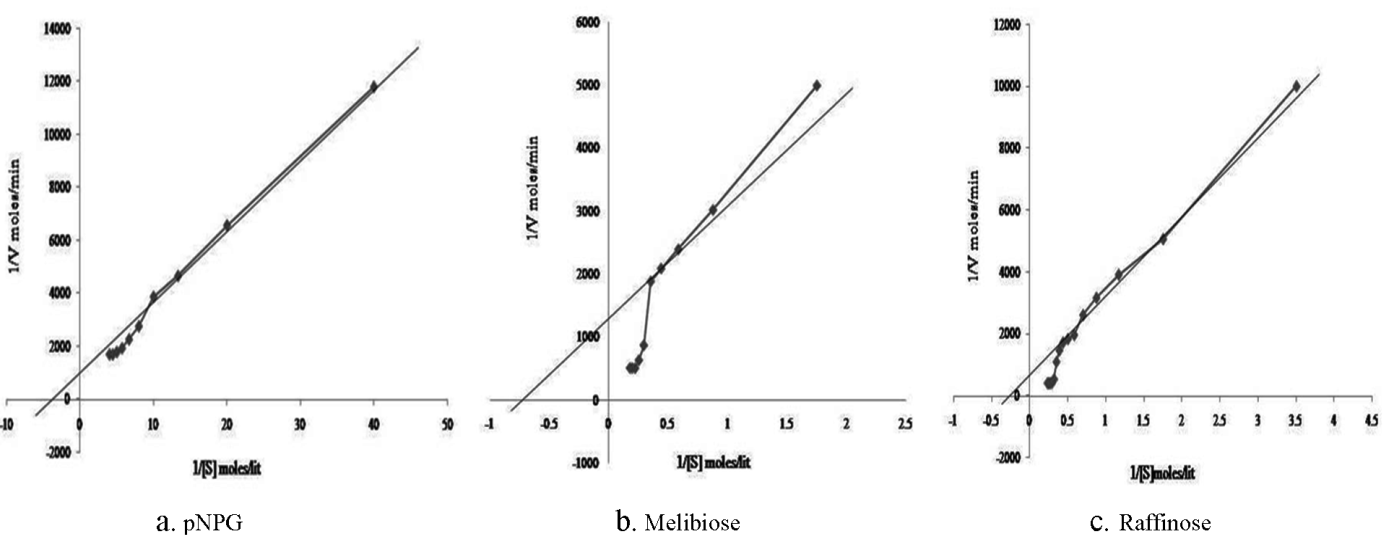


**Fig. 5** Lineweaver–Burk Plot for α-galactosidase from *Fusarium moniliforme* examined for various concentrations of a. pNPG, b. Melibiose and c. Raffinose as a substrate

**Table 1** Representing the coded values in low (-), zero (0) and high (+) levels

| Independent variables | Coded symbols | Low(-) | Zero(0) | High(+) |
| --- | --- | --- | --- | --- |
| Wheat bran (g) | A | 3 | 4 | 5 |
| Peptone (μg) | B | 5 | 252.5 | 500 |
| FeSO_4_.7H_2_O (μg) | C | 0.1 | 5.05 | 10 |
| MgSO_4_.7H_2_O (μg) | D | 5 | 252.5 | 500 |
| pH | E | 5 | 7 | 9 |
| Inoculum size (%) | F | 1 | 3 | 5 |
| Inoculum age (h) | G | 24 | 48 | 72 |
| Incubation period (h) | H | 72 | 96 | 120 |
| Moisture content (%) | I | 50 | 55 | 60 |

**Table 2** Coded variables screened for five levels (- α, -, 0, +, and +α)

| Independent variables | Coded symbol | -α | Low (-) | 0 | High (+) | α |
| --- | --- | --- | --- | --- | --- | --- |
| Wheat bran | A | 2 | 2.810 | 4 | 5.189 | 6 |
| Peptone | B | 0.5 | 152.42 | 375.25 | 598.077 | 750 |
| FeSO_4_.7H_2_O | C | 0.01 | 4.061 | 10.005 | 15.948 | 20 |

**Table 3** Effect of metal ions and EDTA, ß-mercaptoethanol and galactose on the activity of α-galactosidase

| Additives | Relative activity (%) |
| --- | --- |
| Control | 100 ± 1.40 |
| Cu^2+^ | 89.55 ± 4.023 |
| Mg2^+^ | 109.61 ± 2.21 |
| Ca2^+^ | 100.85 ± 1.06 |
| Ba^+^ | 100.05 ± 1.40 |
| Hg^+^ | 0 |
| Na^+^ | 99.91 ± 0.24 |
| K^+^  EDTA  ß-mercaptoethanol  Urea  Galactose (100 mM) | 99.65 ± 4.04  100.05 ± 2.15  100.45 ± 3.77  61.28 ± 3.13  91.94 ± 3.80 |

The activity of α-galactosidase y in the presence of the additives (1mM) was compared with the control whose activity was taken as 100 %. Reaction conditions: Substrate PNPG; Temperature 50°C; pH 4.0. The values are mean ± standard deviation, n=3

**Table 4** Effect of organic solvents on the activity and stability of the purified α-galactosidase.

| Organic solvents | Relative activity (%)  48h 96h 144h |
| --- | --- |
| Control | 100 100 100 |
| Ethanol | 97.7± 1.32 95.63 ± 1.85 91.59 ± 4.31 |
| Methanol | 98.75± 0.261 98.17 ± 1.66 93.6 ± 3.64 |
| n-butanol | 97.68± 0.168 95.77 ± 1.85 91.91 ± 3.86 |
| n-propanol | 98.44± 0.523 92.25 ± 3.02 90.93 ± 5.30 |
| Glycerol | 87.86± 0.392 86.62 ± 4.03 79.75 ± 5.79 |
| Ethanol (70%) | 98.3 ± 0.523 97.19 ± 2.11 92.83 ± 3.86 |
| Acetone | 100 100 99.78 ± 0.224 |

The activity of α-galactosidase in the presence of the organic solvents was compared with the control whose activity was taken as 100 %. Reaction conditions: Substrate PNPG; Temperature 50°C; pH 4. The values are mean ± standard deviation, n=3
